# 异甘草素调控LINC01503对肺鳞癌细胞的作用研究

**DOI:** 10.3779/j.issn.1009-3419.2024.102.30

**Published:** 2024-08-20

**Authors:** Mengshi ZHANG, Yishuang CUI, Yihan YAO, Yanlei GE, Junqing GAN, Ye JIN, Guogui SUN

**Affiliations:** ^1^063210 唐山，华北理工大学公共卫生学院（张梦诗，崔逸爽）; ^1^School of Public Health, North China University of Science and Technology, Tangshan 063210, China; ^2^063000 唐山，华北理工大学附属医院（么艺涵，戈艳蕾，甘俊清，孙国贵）; ^2^Hospital Affiliated to North China University of Science and Technology, Tangshan 063000, China; ^3^063000 唐山，唐山市医工融合精准医疗重点实验室（戈艳蕾，甘俊清，金叶，孙国贵）; ^3^Tangshan Key Laboratory of Precision Medicine, Tangshan 063000, China; ^4^063000 唐山，河北省医工融合精准医疗重点实验室（戈艳蕾，甘俊清，金叶，孙国贵）; ^4^Hebei Provincial Key Laboratory of Medical-Industrial Fusion Precision Medicine, Tangshan 063000, China; ^5^063000 唐山，华北理工大学临床医学院（金叶）; ^5^Clinical Medical College, North China University of Science and Technology, Tangshan 063000, China

**Keywords:** 肺肿瘤, 异甘草素, LINC01503, 增殖, 凋亡, 侵袭, 迁移, Lung neoplasms, Isoliquiritigenin, LINC01503, Proliferation, Apoptosis, Invasive, Migration

## Abstract

**背景与目的:**

异甘草素（isoliquiritigenin, ISL）是甘草中重要的药理成分，其具有一系列的生理和药理活性，同时具有显著的抗肿瘤活性，可以作为癌症靶向治疗的一种潜在药物。LINC01503是一种致癌基因，其与多种癌症的恶性生物学过程密切相关。本研究旨在探讨ISL通过调控LINC01503对肺鳞癌细胞增殖、凋亡、侵袭及迁移的影响。

**方法:**

收集2021年1月至2022年12月于唐山市人民医院治疗的肺鳞癌患者和健康人血浆。用实时荧光定量聚合酶链式反应（real-time quantitative polymerase chain reaction, qRT-PCR）检测LINC01503在肺鳞癌血浆、组织及细胞中的表达情况。用不同浓度的ISL处理肺鳞癌细胞24 h，用qRT-PCR检测LINC01503表达情况。将细胞进行分组处理：si-NC组、si-LINC01503组、DMSO（0.1%的二甲基砜）组、ISL组、pc DNA3.1(+)-NC组、pc DNA3.1(+)-LINC01503组、ISL+pc DNA3.1(+)-NC组及ISL+pc DNA3.1(+)-LINC01503组。采用CCK-8法、克隆形成实验、流式细胞术、Transwell实验和划痕实验研究LINC01503对肺鳞癌细胞功能表型的影响。

**结果:**

荧光原位杂交结果显示，肺鳞癌患者组织芯片中，肺鳞癌组织LINC01503的平均荧光强度高于癌旁组织（P<0.05）。肺鳞癌患者血浆中LINC01503的表达高于健康人血浆表达（P<0.05）。敲降LINC01503可以抑制肺鳞癌细胞的增殖、侵袭及迁移并促进调亡（P<0.05）。ISL可以抑制肺鳞癌细胞的增殖、侵袭、迁移并促进调亡（P<0.05）。过表达LINC01503后用ISL进行干预可逆转过表达LINC01503对肺鳞癌细胞的增殖、侵袭及迁移的促进作用及对调亡的抑制作用（P<0.05）。

**结论:**

LINC01503在肺鳞癌中呈高表达，LINC01503可以促进肺鳞癌细胞的增殖、侵袭、迁移并抑制调亡，ISL可以通过调控LINC01503的表达抑制肺鳞癌细胞的增殖、侵袭、迁移，促进肺鳞癌细胞的凋亡。

## Abstract (en)

**Background and objective:**

Isoliquiritigenin (ISL) is an important pharmacological constituent of Glycyrrhiza glabra, which possesses a range of physiological and pharmacological activities, as well as significant antitumor activity, and can be used as a potential drug for targeted cancer therapy. LINC01503 is an oncogene, which has been closely associated with the malignant biological processes of many cancers. The aim of this study was to investigate the effects of ISL on the proliferation, apoptosis, invasion and migration of lung squamous carcinoma cells by regulating LINC01503.

**Methods:**

Plasma was collected from lung squamous carcinoma patients and healthy individuals treated at Tangshan People's Hospital from January 2021 to December 2022. The expression of LINC01503 in lung squamous carcinoma plasma, tissues and cells was detected by real-time quantitative fluorescence polymerase chain reaction (qRT-PCR). Lung squamous carcinoma cells were treated with different concentrations of ISL for 24 h, and LINC01503 expression was detected by qRT-PCR. The cells were treated in groups: si-NC group, si-LINC01503 group, DMSO (0.1% dimethyl sulfone) group, ISL group, pc DNA3.1(+)-NC group, pc DNA3.1(+)-LINC01503 group, ISL+pc DNA3.1(+)-NC group and ISL+pc DNA3.1(+)- LINC01503 groups. CCK-8 assay, clone formation assay, flow cytometry, Transwell assay and scratch assay were used to explore the effect of LINC01503 on the functional phenotype of lung squamous carcinoma cells.

**Results:**

Fluorescence in situ hybridization results showed that the average fluorescence intensity of LINC01503 in tissue microarrays of lung squamous carcinoma patients was higher than that in paracancerous tissues (P<0.05). The expression of LINC01503 in the plasma of patients with lung squamous carcinoma was higher than that in the plasma of healthy individuals (P<0.05). Knockdown of LINC01503 inhibited the proliferation, invasion and migration of lung squamous carcinoma cells and promoted apoptosis (P<0.05). ISL inhibited the proliferation, invasion, migration and promoted apoptosis of lung squamous carcinoma cells (P<0.05). Overexpression of LINC01503 followed by intervention with ISL reversed the promotional effect of overexpression of LINC01503 on the proliferation, invasion and migration of lung squamous carcinoma cells as well as the inhibitory effect on apoptosis (P<0.05).

**Conclusion:**

LINC01503 was highly expressed in lung squamous carcinoma, and LINC01503 could promote the proliferation, invasion and migration of lung squamous carcinoma cells and inhibit the apoptosis, ISL could inhibit the proliferation, invasion and migration of lung squamous carcinoma cells and promote apoptosis of lung squamous carcinoma cells by regulating the expression of LINC01503.

肺癌是世界范围内主要的五种恶性肿瘤之一，同时也是我国最常见且死亡率最高的恶性肿瘤^[1]^。有研究^[2]^表明，2022年中国恶性肿瘤新发病例及死亡病例中，肺癌位于首位（新发病例106.06万、死亡例数73.33万）。肺癌主要的组织学亚型分为小细胞肺癌（small cell lung cancer, SCLC）和非小细胞肺癌（non-small cell lung cancer, NSCLC）两种，NSCLC是最常见的类型，占肺癌诊断的80%以上，5年存活率在15%左右^[3]^。肺鳞癌属于NSCLC，其约占所有肺癌的40%^[4,5]^。目前，针对NSCLC，化疗、放射治疗、介入治疗、手术治疗、分子靶向治疗^[6]^、免疫疗法^[7]^等都是临床上常用的治疗方法。但是，在目前的研究中，与肺腺癌相比，肺鳞癌几乎还未建立靶向驱动突变，其靶向治疗的效果尚不乐观^[8]^。因此，研究肺鳞癌发生发展的内在分子机制，寻找针对肺鳞癌的靶向小分子药物至关重要。

异甘草素（isoliquiritigenin, ISL）是一种从甘草根中提取的天然黄酮类化合物，具有较高的抗肿瘤活性^[9]^。研究^[10⇓⇓⇓-14]^表明，ISL通过调控肿瘤细胞的信号通路、诱导细胞的凋亡和自噬、逆转细胞上皮间充质转化（epithelial-mesenchymal transition, EMT）、抗氧化等机制在乳腺癌、口腔鳞癌、卵巢癌、舌鳞细胞癌等的恶性肿瘤中发挥抗癌作用。有研究^[15]^表明，ISL可以通过靶向miR-301b及其靶基因而抑制黑色素瘤细胞的增殖和凋亡，同时在体内实验证实ISL能通过调控miRNA来抑制肿瘤的生长，从而证明ISL可以靶向调控miRNA而发挥抗肿瘤作用。现有研究^[16]^表明，ISL可以通过其代谢产物2,4,2′,4′-四羟基查耳酮（THC）抑制肺癌细胞内Src酪氨酸激酶的活性从而阻止细胞迁移并取消细胞骨架重组及黏着斑组装，抑制肺癌的发展。此外，ISL可以通过靶向胰岛素样生长因子-2 mRNA结合蛋白3（insulin-like growth factor-2 mRNA-binding proteins 3, IGF2BP3）/N6-甲基腺苷（N6-methyladenosine, m6A）/Twist家族bHLH转录因子1（Twist family bHLH transcription factor 1, TWIST1）轴抑制NSCLC细胞的增殖、迁移和侵袭^[17]^。在肺腺癌中，ISL通过抑制EMT过程抑制A549细胞的迁移和侵袭，并通过激活磷脂酰肌醇-3激酶（phosphatidylinositol 3-kinase, PI3K）/蛋白激酶B（protein kinase B, PKB）信号通路抑制肺癌细胞的生长并促进其凋亡^[18]^。

长链非编码RNA（long non-coding RNAs, lncRNAs）是一类不具备蛋白编码能力且长度大于200个核苷酸的RNA转录本，其异常表达可以介导癌症及其他疾病的发展。研究^[19]^表明，lncRNAs广泛参与肿瘤细胞的生长发展过程，如增殖、侵袭、迁移等。LINC01503首次在侵袭性鳞状细胞癌中被发现是一种致癌基因。研究^[20⇓⇓⇓-24]^发现，LINC01503与部分恶性肿瘤的发展密切相关。研究^[25,26]^证实，LINC01503在肺腺癌中可以通过调控其转录因子或与miRNA结合促进其进展和转移。但是目前尚无有关LINC01503在肺鳞癌中的研究。因此，本研究主要探究ISL通过调控LINC01503的表达对肺鳞癌细胞增殖、凋亡、侵袭及迁移能力的影响，从而为肺鳞癌的治疗提供新选择。

## 1 材料与方法

### 1.1 材料

#### 1.1.1 血浆来源

选取2021年1月至2022年12月于唐山市人民医院就诊的肺鳞癌患者20例并匹配年龄相仿性别相同的健康人20例。本实验由唐山市人民医院伦理委员会批准监督（批准编号：RMYY-LLKS-2021-103）。

#### 1.1.2 细胞和主要试剂

NCI-H1703、NCI-H226和SK-MES-1细胞为人肺鳞状细胞癌细胞系，BEAS-2B为人支气管上皮细胞。NCI-H1703、NCI-H226和SK-MES-1细胞株购于武汉普诺赛生命科技有限公司；BEAS-2B购于北京中生奥邦生物科技有限公司；组织芯片购于上海芯超生物科技有限公司。NEOFECT转染试剂购于北京码因科技有限公司；Lipofectamine 2000转染试剂购于美国Invitrogen公司；Trizol试剂购于美国Ambion公司；lncRNA逆转录试剂盒和荧光定量聚合酶链式反应（real-time quantitative polymerase chain reaction, qRT-PCR）检测试剂盒均购自日本Takara公司；LINC01503过表达质粒均购于北京西贝宏程生物科技有限公司；LINC01503荧光原位杂交探针（fluorescence in situ hybridization, FISH）、LINC01503引物、LINC01503的小干扰RNA（si-LINC01503）、乱序无意义阴性序列（si-NC）及FISH试剂盒购于广州市锐博生物科技有限公司；细胞计数试剂盒8（cell counting kit-8, CCK-8）购于北京中生奥邦生物科技有限公司。

### 1.2 方法

#### 1.2.1 细胞培养

将肺鳞癌细胞NCI-H1703和NCI-H226用含10%的胎牛血清、1%青霉素-链霉素的1640培养基放入T25培养瓶内，在37 ^o^C、5% CO_2_的培养箱内进行培养，待细胞密度达到80%-90%即可进行传代、冻存和后续实验。

#### 1.2.2 细胞转染和分组

取对数生长期的H1703和H226细胞，使用Lipofectamine 2000试剂盒按照说明书将si-NC、si-LINC01503转染至铺于6孔板内的细胞中，将细胞分为si-NC组和si-LINC01503组。同时，使用二甲基砜（dimethyl sulphoxide, DMSO; 0.1%）及ISL（50 μmol/L）将细胞分为DMSO组及ISL组。为了探究ISL可以通过调控LINC01503而影响肺鳞癌细胞的增殖、凋亡、侵袭及迁移能力，用质粒转染试剂转染pcDNA 3.1(+)-NC及pcDNA 3.1(+)-LINC01503或使用ISL（50 μmol/L）处理，将细胞分为pcDNA 3.1(+)-NC组、pcDNA 3.1(+)-LINC01503组、ISL+pcDNA 3.1(+)-NC组及ISL+pcDNA 3.1(+)-LINC01503组。常规培养24 h后进行后续实验。

#### 1.2.3 实时荧光定量PCR（real-time quantitative PCR, qRT-PCR）

采用通用型RNA提取试剂盒提取血浆和细胞总RNA，根据逆转录说明书将提取的RNA逆转录为cDNA，根据实时荧光定量试剂盒加样后上机检测LINC01503的表达。LINC01503上游引物序列为5'-TCTTCGTGTTCATCATCAGTCCC-3'，下游引物序列为5'-CTGAAAGAAACTCATTGCATCGTG-3'。GAPDH上游引物序列为5'-ACCCACTCCTCCACCTTTGA-3'，下游引物序列为5'-CCACCCTGTTGCTGTAGCCA-3'。以GADPH为内参，检测LINC01503反应条件为95 ^o^C、30 s，95 ^o^C、3 s，60 ^o^C、30 s，40个循环。采用2^-△△Ct^法计算LINC01503的相对表达量。

#### 1.2.4 CCK-8法检测细胞增殖活性

按照实验分组对细胞进行转染，24 h后将细胞消化离心并进行计数，按2×10^3^个/孔的细胞量接种于96孔板（100 μL/孔），每组设置5个复孔，在培养箱中继续培养24、48、72和96 h，每孔加入10 μL CCK-8溶液，2 h后用酶标仪于450 nm波长处测吸光度值。

#### 1.2.5 克隆形成实验检测细胞增殖能力

将细胞分组处理后进行消化、离心、重悬计数，取3×10^3^个细胞铺在12孔板内，在培养箱内培养14 d，每隔3 d换液1次。培养结束后弃去旧培养基，用甲醇固定10 min后用0.1%的结晶紫进行染色，拍照计算细胞数。

#### 1.2.6 Transwell实验检测细胞侵袭迁移

将细胞分组处理后进行消化、离心，用双无培养基进行重悬计数。提前将50 μL稀释后的Matrigel胶（Matrigel胶:双无培养基=1:8）加入小室的上室，取150 μL含8×10^4^个细胞的细胞悬液加入上室（200 μL/孔），下室内加入600 μL高血清（含30%胎牛血清）培养基，置于培养箱中培养24 h后，用甲醇固定10 min后用0.1%的结晶紫进行染色，在倒置光学显微镜下进行拍照，计算穿过细胞数。迁移实验无需加Matrigel胶，其余步骤与侵袭实验步骤相同。

#### 1.2.7 划痕实验检测细胞迁移

将细胞分组处理后铺于6孔板内进行培养，待细胞密度达到80%-90%时用20 μL的枪头在6孔板底部垂直划出一条直线，用生理盐水清洗干净后加入低血清培养基（含2%胎牛血清），分别在0和24 h置于显微镜下进行拍照。使用Image J测量迁移面积，计算迁移率。

#### 1.2.8 流式细胞术检测细胞凋亡

将细胞分组处理后进行消化、离心后弃去上清，每组细胞沉淀内加入1×Binding buffer混匀，后吸取100 μL至EP管内，分别避光加入5 μL FITC Annexin V溶液及5 μL的PI溶液，再加入400 μL的1×Binding buffer混匀，用流式细胞仪进行上机检测。

#### 1.2.9 荧光原位杂交

细胞：将2×10^4^个/孔的细胞量置于24孔板内的爬片上，培养24 h进后行细胞固定及通透，然后加入预杂交液37 ^o^C封闭30 min。避光条件下，把2.5 μm探针（20 μmol/L）加入100 μL的杂交液（37 ^o^C预热）中，获得探针杂交液。弃去每孔细胞中的预杂交液，加入制备的探针杂交液，避光，37 ^o^C杂交16 h，后用提前42 ^o^C预热好的杂交洗液I、II、III，避光洗涤细胞。最后用PBS洗涤细胞，将细胞爬片取出用含有DAPI的封片剂封片。组织：将组织芯片放入烤箱60 ^o^C、2 h进行脱蜡，将组织芯片放入二甲苯I（脱蜡用）20 min→二甲苯II 20 min→100%酒精I（脱二甲苯用）10 min→100%酒精II 10 min→90%酒精5 min→80%酒精5 min→70%酒精5 min→自来水5 min；3% H_2_O_2_室温处理10 min以灭活内源性过氧化物酶，蒸馏水洗涤5 min，共2次；切片上滴加3%柠檬酸新鲜稀释的胃蛋白酶（1 mL 3%柠檬酸加2滴浓缩型胃蛋白酶，混匀），37 ^o^C消化15 min。0.5 mol/L PBS洗涤5 min，共3次；蒸馏水洗涤5 min，共1次；然后加入预杂交液37 ^o^C封闭30 min。避光条件下，把2.5 μL含LINC01503的荧光探针（20 μmol/L）加入100 μL的杂交液（37 ^o^C预热）中，获得探针杂交液。弃去预杂交液，LINC01503组加入制备好的探针杂交液，空白组加入不含探针的杂交液，避光，37 ^o^C杂交16 h。其余步骤同细胞。

### 1.3 统计学分析

采用GraphPad Prism 9.0统计分析软件对所得数据进行分析及图像绘制，计量资料以均数±标准差表示，组间比较采用两独立样本t检验或单因素方差分析，计数资料的比较采用卡方检验，P<0.05为差异有统计学意义。

## 2 结果

### 2.1 LINC01503在肺鳞癌组织和血浆中的表达情况

采用荧光原位杂交实验对LINC01503在肺鳞癌患者的组织芯片中的表达进行荧光强度分析。结果显示，在肺鳞癌患者组织芯片中，相对于癌旁组织，癌组织中的平均荧光强度显著提高（P<0.0001，图1A、1B）。qRT-PCR结果显示，在肺鳞癌患者的血浆中，LINC01503的表达水平高于健康人的血浆中的表达（P<0.0001，图1C）。

**图1:**
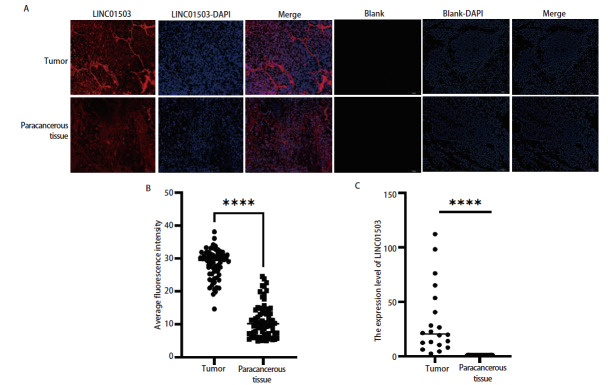
LINC01503在肺鳞癌组织芯片（×200）及血浆中的表达情况。A、B：荧光原位杂交实验验证肺鳞癌组织及癌旁组织中LINC01503表达情况。C：qRT-PCR检测LINC01503在健康人及肺鳞癌患者血浆中的表达情况。****P<0.0001。

### 2.2 LINC10503在肺鳞癌细胞系中的表达及定位

qRT-PCR实验结果显示，相对于正常的肺上皮细胞BEAS-2B，在肺鳞癌细胞NCI-H1703和NCI-H226中，LINC10503的表达水平均呈现出高表达趋势（P<0.05、0.01，图2A）。此外，荧光原位杂交及核质分离结果显示，LINC01503主要定位于细胞质中（图2B）。

**图2:**
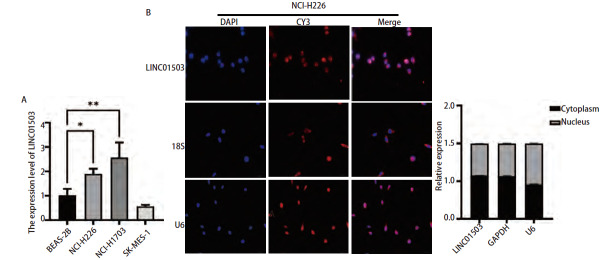
LINC01503在肺鳞癌细胞中的表达及定位。A：LINC01503在肺鳞癌细胞系中的表达情况；B：荧光原位杂交及核质分离实验LINC01503在NCI-H226细胞中的定位。*P<0.05；**P<0.01。

### 2.3 敲低LINC01503对肺鳞癌细胞增殖凋亡能力的影响

将si-NC和si-LINC01503分别转染至肺鳞癌细胞NCI-H1703和NCI-H226中，并命名为si-NC组及si-01503组。采用qRT-PCR技术检测si-NC和si-01503的转染效率，结果显示，si-10503组NCI-H1703及NCI-H226细胞LINC01503的表达水平分别显著低于si-NC组的表达（均P<0.001，图3A）。CCK-8实验结果显示，敲降LINC01503后，si-01503组NCI-H1703和NCI-H226细胞活力相比于si-NC组更低（均P<0.0001，图3B）。克隆结果显示，si-01503组NCI-H1703，NCI-H226细胞的克隆细胞数显著低于si-NC组（均P<0.01，图3C）。流式细胞术结果显示，si-NC组NCI-H1703及NCI-H226细胞的凋亡率均低于si-01503组的细胞凋亡率（P<0.0001、0.01，图3D）。以上结果表明，敲降LINC01503可以抑制肺鳞癌细胞的增殖，促进其调亡。

**图3:**
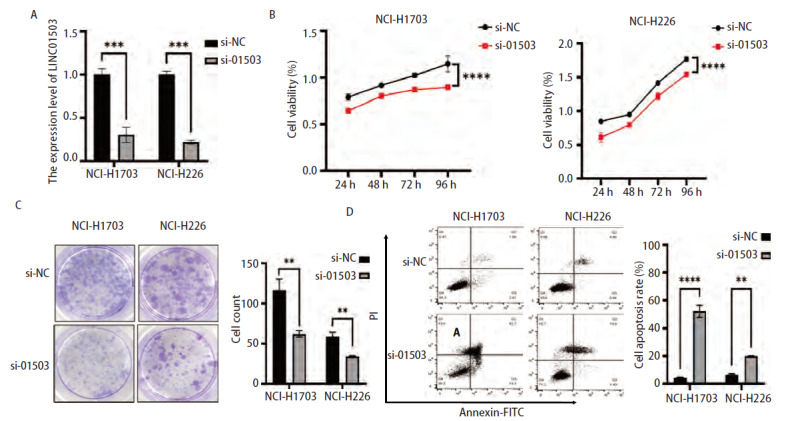
敲降LINC01503抑制肺鳞癌细胞NCI-H1703和NCI-H226细胞增殖并促进凋亡。A：qRT-PCR实验检测敲降LINC01503后肺鳞癌细胞中LINC01503表达情况；B：CCK-8实验检测敲降LINC01503后细胞增殖能力；C：克隆形成实验检测敲降LINC01503后细胞增殖能力；D：流式细胞术检测敲降LINC01503后细胞调亡能力。**P<0.01；***P<0.001；****P<0.0001。

### 2.4 敲低LINC01503对肺鳞癌细胞侵袭迁移能力的影响

Transwell实验结果显示，si-01503组NCI-H1703及NCI-H226细胞的侵袭数均小于si-NC组（均P<0.01，图4A），si-01503组细胞的迁移数也显著低于si-NC组（P<0.05、0.01，图4B）。细胞划痕实验结果显示，si-NC组NCI-H1703及NCI-H226细胞的迁移率明显高于si-01503组细胞的迁移率（P<0.05、0.01，图4C）。以上结果表明，敲降LINC01503可以抑制肺鳞癌细胞的侵袭及迁移。

**图4:**
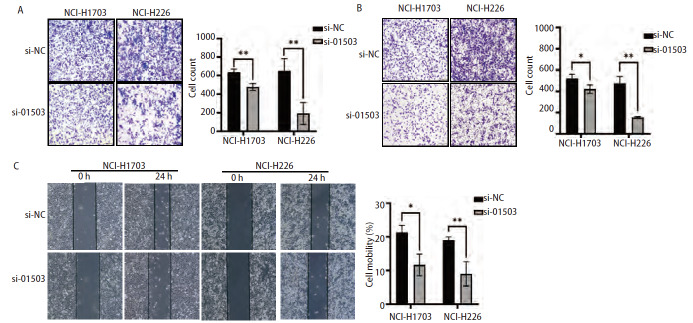
敲降LINC01503抑制肺鳞癌细胞侵袭迁移能力。A：Transwell实验检测敲降LINC01503后细胞侵袭能力；B：Transwell实验检测敲降LINC01503后细胞迁移能力；C：划痕实验检测敲降LINC01503后细胞迁移能力。*P<0.05；**P<0.01。

### 2.5 ISL对肺鳞癌细胞NCI-H1703和NCI-H226活力的影响

采用不同浓度的ISL（0、20、40、60、80、100 μmol/L）处理NCI-H1703和NCI-H226细胞，24 h后采用CCK-8实验检测其细胞存活率。结果显示，ISL以剂量依赖性抑制NCI-H1703和NCI-H226细胞的细胞存活能力（P<0.05，图5）。ISL对NCI-H1703的半抑制浓度（half maximal inhibitory concentration, IC_50_）值为49.89 μmol/L，NCI-H226的IC_50_值为54.96 μmol/L，在后续实验中，我们选择50 μmol/L的ISL浓度对肺鳞癌细胞进行处理。

**图5:**
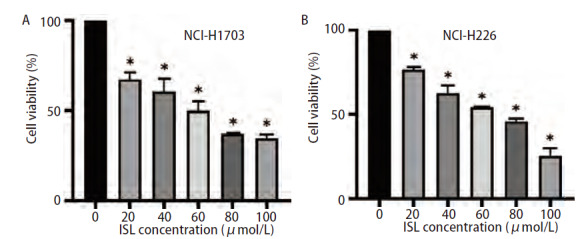
不同浓度ISL对肺鳞癌细胞NCI-H1703（A）和NCI-H226（B）存活率的影响。与ISL（0 μmol/L）组相比，P<0.05。

### 2.6 ISL对肺鳞癌细胞增殖及凋亡能力的影响

采用不同浓度的ISL（0、20、40、60、80、100 μmol/L）处理NCI-H1703及NCI-H226细胞，24 h后采用qRT-PCR实验检测LINC01503的表达情况，结果显示，LINC01503的表达随着ISL浓度的增高而降低（P<0.05，图6A）。qRT-PCR结果显示，ISL（50 μmol/L）处理后在BEAS-2B、NCI-H1703及NCI-H226中LINC01503的表达水平均为低于DMSO组LINC01503的表达水平（P<0.001、0.01、0.05，图6B）。CCK-8结果显示，培养24、48、72、96 h后，DMSO组的细胞活力均高于ISL组（均P<0.0001，图6C）。克隆结果显示，ISL处理后BEAS-2B、NCI-H1703及NCI-H226细胞的克隆细胞数显著低于DMSO组（P<0.01、0.05、0.01，图6D）。流式细胞术结果显示，DMSO组细胞的凋亡率均低于ISL组（P<0.05、0.01、0.001，图6E）。以上结果表明，ISL可以抑制肺鳞癌细胞的增殖，促进其调亡。

**图6:**
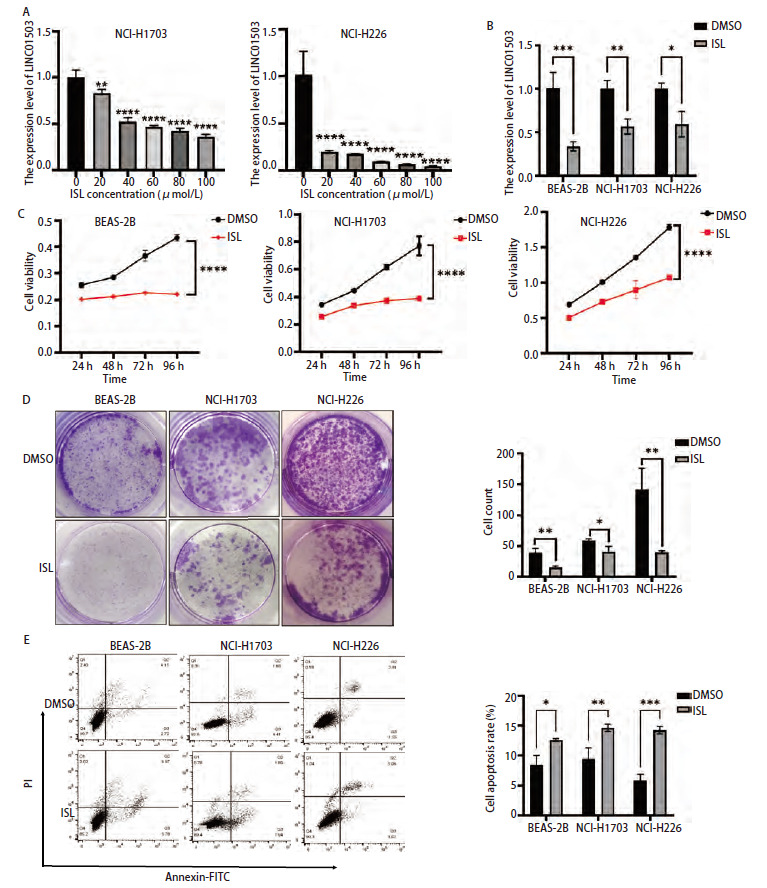
ISL可以抑制肺鳞癌细胞NCI-H1703和NCI-H226细胞增殖并促进凋亡。A：不同浓度ISL处理后PCR实验检测LINC01503的表达情况；B：qRT-PCR实验检测ISL（50 μmol）处理后肺鳞癌细胞中LINC01503表达情况；C、D：ISL处理后CCK-8实验检测细胞增殖能力；E：ISL处理后采用流式细胞术检测细胞的调亡能力。*P<0.05；**P<0.01；***P<0.001；****P<0.0001。

### 2.7 ISL对肺鳞癌细胞侵袭迁移能力的影响

Transwell结果显示，DMSO组BEAS-2B、NCI-H1703和NCI-H226细胞的侵袭数均高于ISL组（P<0.01、0.05、0.001，图7A），DMSO组细胞的迁移数均高于ISL组（P<0.001、0.001、0.05，图7B）。划痕实验结果显示，ISL组细胞的迁移率明显较DMSO组低（均P<0.01，图7C）。以上结果表明，ISL可以抑制肺鳞癌细胞的侵袭迁移能力。

**图7:**
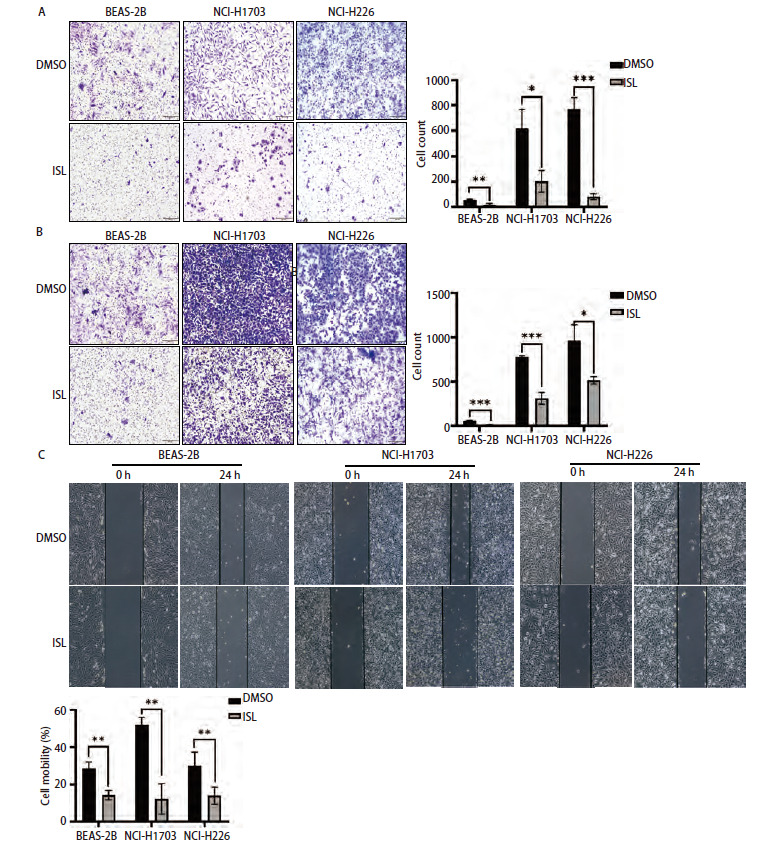
ISL抑制肺鳞癌细胞侵袭迁移能力。A：Transwell实验检测ISL处理后细胞侵袭能力；B：Transwell实验检测ISL处理后细胞迁移能力；C：划痕实验检测ISL处理后细胞迁移能力。*P<0.05；**P<0.01；***P<0.001。

### 2.8 ISL调控LINC01503对肺鳞癌细胞增殖凋亡、侵袭迁移能力的影响

将过表达LINC01503质粒pcDNA 3.1(+)-NC和pcDNA 3.1(+)-LINC01503转染至肺鳞癌细胞NCI-H1703和NCI-H226中，并命名为pcDNA 3.1(+)-NC组和pcDNA 3.1(+)+LINC01503组，同时将过表达LINC01503联合ISL共同作用肺鳞癌细胞NCI-H1703及NCI-H226，命名为ISL+pcDNA 3.1(+)-NC组和ISL+pcDNA 3.1(+)+LINC01503组。CCK-8实验结果显示，在培养24、48、72、96 h后，pcDNA 3.1(+)-NC组NCI-H1703及NCI-H226细胞的活力高于ISL+pcDNA 3.1(+)-NC组（P<0.001、0.01），pcDNA 3.1(+)-LINC01503组的细胞活力显著高于ISL+pcDNA 3.1(+)-LINC01503组（均P<0.05，图8A）。克隆实验结果显示，pcDNA 3.1(+)-NC组NCI-H1703及NCI-H226细胞的细胞克隆数均高于ISL+pcDNA 3.1(+)-NC组（P<0.001、0.0001），pcDNA 3.1(+)-LINC01503组NCI-H1703及NCI-H226细胞克隆数显著高于ISL+pcDNA 3.1(+)-LINC01503组（均P<0.0001，图8B）。流式细胞术结果显示，pcDNA 3.1(+)-NC组细胞凋亡率均低于ISL+pcDNA 3.1(+)-NC组（P<0.05、0.01），pcDNA 3.1(+)-LINC01503组细胞的凋亡率均低于ISL+ pcDNA 3.1(+)-LINC01503组的（P<0.01、0.05，图9）。Transwell实验显示，pcDNA 3.1(+)-NC组NCI-H1703及NCI-H226细胞的侵袭数均高于ISL+pcDNA 3.1(+)-NC组（均P<0.001），pcDNA 3.1(+)-LINC01503组细胞侵袭数均高于ISL+pcDNA 3.1(+)-LINC01503组（P<0.001、0.0001，图10A）。此外，在pcDNA 3.1(+)-NC组中，NCI-H1703及NCI-H226细胞的迁移数均高于ISL+pcDNA 3.1(+)-NC组（P<0.0001、0.05），pcDNA 3.1(+)-LINC01503组细胞的迁移数均高于ISL+pcDNA 3.1(+)-LINC01503组（P<0.0001、0.05，图10B）。在划痕实验中，pcDNA 3.1(+)-NC组NCI-H1703及NCI-H226细胞的迁移率均高于ISL+pcDNA 3.1(+)-NC组（P<0.001、0.05），pcDNA 3.1(+)-LINC01503组细胞的迁移率均高于ISL+pcDNA 3.1(+)-LINC01503组（P<0.0001、0.01，图11）。以上结果表明，ISL可以逆转过表达LINC01503对肺鳞癌细胞增殖、侵袭迁移及调亡能力的影响。

**图8:**
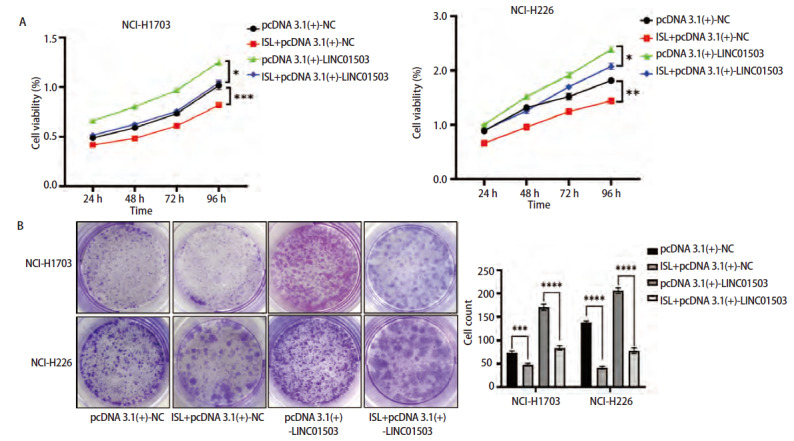
ISL逆转过表达LINC01503对肺鳞癌细胞增殖能力的影响。A：CCK-8实验检测过表达LINC01503后ISL处理对细胞增殖能力的影响；B：克隆形成实验检测过表达LINC01503后ISL处理对细胞增殖能力的影响。*P<0.05；**P<0.01；***P<0.001；****P<0.0001。

**图9:**
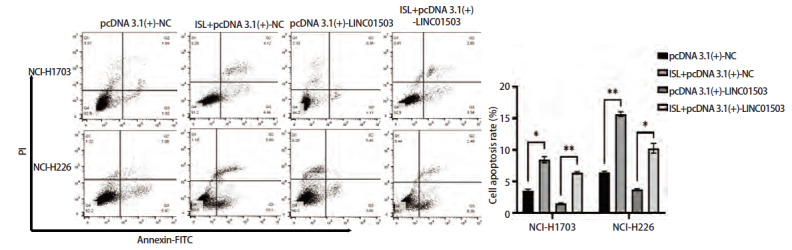
流式细胞术检测ISL逆转过表达LINC01503对肺鳞癌细胞凋亡能力的影响。*P<0.05；**P<0.01。

**图10:**
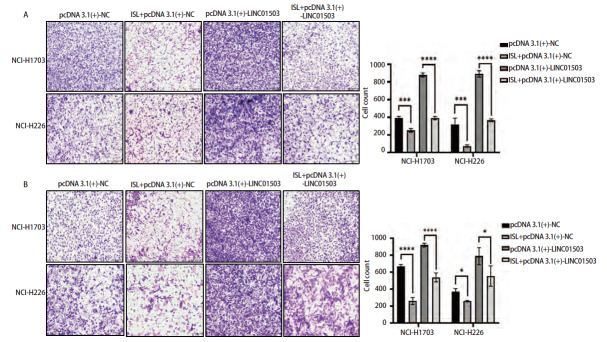
Transwell实验验证ISL逆转过表达LINC01503对肺鳞癌细胞侵袭迁移能力的影响。A：Transwell实验验证过表达LINC01503后ISL处理对NCI-H1703及NCI-H226细胞侵袭能力的影响；B：Transwell实验验证过表达LINC01503后ISL处理对细胞迁移能力的影响。**P<0.05；***P<0.001；****P<0.0001。

**图11:**
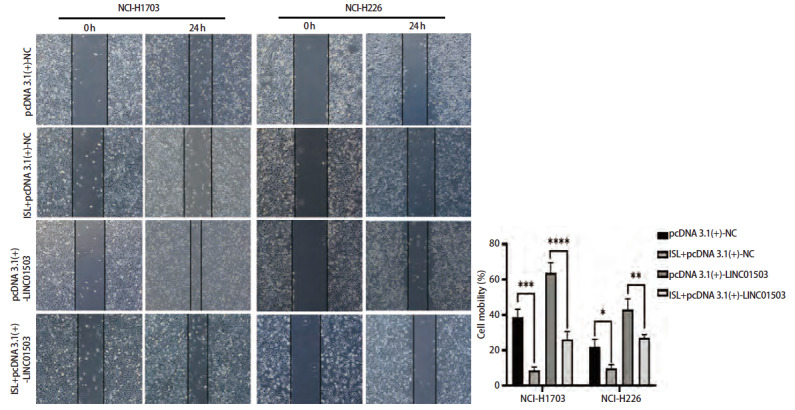
划痕实验验证ISL逆转过表达LINC01503对肺鳞癌细胞迁移能力的影响。*P<0.05；**P<0.01；***P<0.001；****P<0.0001。

## 3 讨论

ISL是从甘草根中提取的一种天然黄酮类化合物，具有抗炎^[27]^、抗氧化^[28]^、抗肿瘤^[29,30]^、抗动脉粥样硬化^[31]^等一系列显著的生物及药理活性。近年来，关于ISL在各种癌症发生发展过程中作用的研究也越来越多。Wang等^[32]^发现ISL在人肝细胞癌HepG2细胞中通过增加活性氧（reactive oxygen species, ROS）水平调节丝裂原活化蛋白激酶（mitogen-activated protein kinase, MAPK）/信号转导和转录激活因子3（signal transducer and activator of transcription 3, STAT3）/核因子κB（nuclear factor-kappa B, NF-κB）通路相关蛋白的表达，导致G_2_/M期细胞周期阻滞和细胞生长抑制。Chen等^[33]^研究发现，ISL通过抑制线粒体蛋白mitoNEET的表达，使ISL介导的ROS含量增加，从而诱导线粒体功能障碍和凋亡。Huang等^[34]^通过研究证明ISL可以在体内抑制Hep3B细胞的增殖和转移。本实验主要研究ISL对肺鳞癌细胞及支气管上皮细胞增殖、侵袭、迁移及凋亡能力的影响。结果显示，与对照DMSO组相比，ISL可以抑制肺鳞癌细胞的增殖、侵袭、迁移并促进肺鳞癌细胞的凋亡。

随着高通量测序技术的快速发展，大量lncRNAs被挖掘，lncRNAs作为一些生物调控过程中重要的调节因子，其调节机制也被广泛关注。LINC01503在肿瘤组织中的表达上调，并且在多种肿瘤中起致癌作用，与各种癌症的发生、预后及转移有关，如胃癌^[20]^、胆管癌^[21]^、宫颈癌^[22]^、肝细胞癌^[24]^等。本研究中LINC01503在肺鳞癌细胞，血浆及组织中都呈高表达。高表达LINC01503的肺鳞癌患者肿瘤转移风险高且生存期较差，是肺鳞癌患者预后不良的一种潜在危险因素。通过分析肺鳞癌患者组织及血清中LINC01503的表达水平，可以评估肿瘤的预后，包括预测患者的生存期和疾病进展的风险，这提示LINC01503作为一种预后指标具有巨大潜力。此外，本研究表明，敲降LINC01503后可以抑制肺鳞癌细胞的增殖、侵袭、迁移并抑制其调亡。有研究显示ISL可以通过调控miRNA、lncRNA等介导癌症的发生发展过程。如：Wang等^[35]^经研究发现胶质瘤细胞中过表达lncRNA NEAT1可以逆转ISL介导的miR-194-5p表达的增加，从而抑制与血管生成相关的蛋白激酶B-成纤维细胞因子-2（protein kinase B-fibroblast growth factor-2, PKB-FGF-2）/转化生长因子-β（transforming growth factor-β, TGF-β）/血管内皮生长因子（vascular endothelial growth factor, VEGF）信号通路。在本研究中，采用不同浓度ISL处理肺鳞癌细胞NCI-H1703及NCI-H226，发现肺鳞癌细胞的存活率随着ISL的浓度升高而下降。此外，随着ISL浓度的升高，LINC01503的表达也呈下降趋势。在肺鳞癌细胞中转染过表达LINC10503质粒后用ISL进行干预，可以逆转过表达LINC10503对肺鳞癌细胞的增殖、侵袭、迁移、凋亡能力的影响。说明ISL通过调节LINC10503的表达水平可以抑制肺癌细胞的增殖、侵袭和迁移，且促进凋亡。

综上所述，该研究初步证明了LINC01503在肺鳞癌细胞、组织、血浆中呈高表达。ISL通过调控LINC01503的表达从而抑制肺鳞癌细胞的增殖、侵袭、迁移并促进凋亡。此实验结果为ISL应用于肺鳞癌的治疗及LINC01503作为肺鳞癌治疗靶点提供了实验依据。

## References

[1] Chi Y, Wang D, Wang J, et al. Long non-coding RNA in the pathogenesis of cancers. Cells, 2019, 8(9): 1015. doi: 10.3390/cells8091015 PMC6770362 31480503

[2] Pan Y, Han H, Labbe KE, et al. Recent advances in preclinical models for lung squamous cell carcinoma. Oncogene, 2021, 40(16): 2817-2829. doi: 10.1038/s41388-021-01723-7 33707749

[3] Chen Z, Fillmore CM, Hammerman PS, et al. Non-small-cell lung cancers: a heterogeneous set of diseases. Nat Rev Cancer, 2014, 14(8): 535-546. doi: 10.1038/nrc3775 25056707 PMC5712844

[4] Li Y, Gu J, Xu F, et al. Transcriptomic and functional network features of lung squamous cell carcinoma through integrative analysis of GEO and TCGA data. Sci Rep, 2018, 8(1): 15834. doi: 10.1038/s41598-018-34160-w PMC6203807 30367091

[5] Gao L, Guo YN, Zeng JH, et al. The expression, significance and function of cancer susceptibility candidate 9 in lung squamous cell carcinoma: A bioinformatics and in vitro investigation. Int J Oncol, 2019, 54(5): 1651-1664. doi: 10.3892/ijo.2019.4758 30896821 PMC6439977

[6] Li JM, Yao XY, Qiu LJ, et al. ICIs combination therapy applied to non-small cell lung cancer after resistance to EGFR mutation-targeted therapy. Zhongguo Feiai Zazhi, 2023, 26(5): 392-399. 37316449 10.3779/j.issn.1009-3419.2023.101.17 PMC10273152

[7] Liu YY, Miao JL. Progress of immunotherapy in advanced non-small cell lung cancer with EGFR mutation. Zhongguo Feiai Zazhi, 2023, 26(12): 934-942. 10.3779/j.issn.1009-3419.2023.106.26 PMC10767652 38163979

[8] Pan Y, Han H, Labbe KE, et al. Recent advances in preclinical models for lung squamous cell carcinoma. Oncogene, 2021, 40(16): 2817-2829. doi: 10.1038/s41388-021-01723-7 33707749

[9] Xu Z, Chen YC, Lin LF, et al. Research progress of new anti-tumor drug delivery systems for isoglycyrrhizin. Zhongcaoyao, 2023, 54(17): 5806-5815.

[10] Zheng H, Li Y, Wang Y, et al. Downregulation of COX-2 and CYP 4A signaling by isoliquiritigenin inhibits human breast cancer metastasis through preventing anoikis resistance, migration and invasion. Toxicol Appl Pharmacol, 2014, 280(1): 10-20. doi: 10.1016/j.taap.2014.07.018 25094029

[11] Hsia SM, Yu CC, Shih YH, et al. Isoliquiritigenin as a cause of DNA damage and inhibitor of ataxia-telangiectasia mutated expression leading to G_2_/M phase arrest and apoptosis in oral squamous cell carcinoma. Head Neck, 2016, 38 Suppl 1: E360-E371. doi: 10.1002/hed.24001 25580586

[12] Hou C, Li W, Li Z, et al. Synthetic isoliquiritigenin inhibits human tongue squamous carcinoma cells through its antioxidant mechanism. Oxid Med Cell Longev, 2017, 2017: 1379430. doi: 10.1155/2017/1379430 28203317 PMC5292127

[13] Chen C, Huang S, Chen CL, et al. Isoliquiritigenin inhibits ovarian cancer metastasis by reversing epithelial-to-mesenchymal transition. Molecules, 2019, 24(20): 3725. doi: 10.3390/molecules24203725 PMC6833095 31623144

[14] Chen H Y, Huang TC, Shieh TM, et al. Isoliquiritigenin induces autophagy and inhibits ovarian cancer cell growth. Int J Mol Sci, 2017, 18(10): 2025. doi: 10.3390/ijms18102025 PMC5666707 28934130

[15] Xiang S, Chen H, Luo X, et al. Isoliquiritigenin suppresses human melanoma growth by targeting miR-301b/LRIG1 signaling. J Exp Clin Cancer Res, 2018, 37(1): 184. doi: 10.1186/s13046-018-0844-x. PMC6091185 30081934

[16] Chen C, Shenoy AK, Padia R, et al. Suppression of lung cancer progression by isoliquiritigenin through its metabolite 2,4,2', 4'-tetrahydroxychalcone. J Exp Clin Cancer Res, 2018, 37(1): 243. doi: 10.1186/s13046-018-0902-4 PMC6171243 30285892

[17] Cui Y, Wu Y, Wang C, et al. Isoliquiritigenin inhibits non-small cell lung cancer progression via m^6^A/IGF2BP3-dependent TWIST 1 mRNA stabilization. Phytomedicine, 2022, 104: 154299. doi: 10.1016/j.phymed.2022.154299 35816995

[18] Tian T, Sun J, Wang J, et al. Isoliquiritigenin inhibits cell proliferation and migration through the PI3K/AKT signaling pathway in A 549 lung cancer cells. Oncol Lett, 2018, 16(5): 6133-6139. doi: 10.3892/ol.2018.9344 30344755 PMC6176347

[19] Xie JJ, Jiang YY, Jiang Y, et al. Super-enhancer-driven long non-coding RNA LINC01503, regulated by TP63, is over-expressed and oncogenic in squamous cell carcinoma. Gastroenterology, 2018, 154(8): 2137-2151.e1. doi: 10.1053/j.gastro.2018.02.018 29454790

[20] Ma Z, Gao X, Shuai Y, et al. EGR1-mediated linc01503 promotes cell cycle progression and tumorigenesis in gastric cancer. Cell Prolif, 2021, 54(1): e12922. doi: 10.1111/cpr.12922 PMC7791171 33145887

[21] Qu YK, Qu XS, Chen G, et al. LINC01503 promotes cell proliferation, invasion and EMT process in cholangio-carcinoma. Eur Rev Med Pharmacol Sci, 2019, 23(15): 6445-6452. doi: 10.26355/eurrev_201908_18526 31378883

[22] Feng J, Gao FY, Li YY, et al. Upregulation of LINC01503 promotes cervical cancer progression by targeting the miR-615-3p/CCND1 axis. J Cancer, 2021, 12(15): 4552-4560. doi: 10.7150/jca.54148 34149919 PMC8210569

[23] An B, Cai Y, Zhu J, et al. Long noncoding RNA LINC 01503 silencing suppresses KLK4 expression to impede pancreatic cancer development as miR-1321 sponge. Biomed Res Int, 2023, 2023: 5403344. doi: 10.1155/2023/5403344 36785666 PMC9922183

[24] Wang MR, Fang D, Di MP, et al. Long non-coding RNA LINC 01503 promotes the progression of hepatocellular carcinoma via activating MAPK/ERK pathway. Int J Med Sci, 2020, 17(9): 1224-1234. doi: 10.7150/ijms.45256 32547318 PMC7294912

[25] Shen Q, Sun Y, Xu S. LINC01503/miR-342-3p facilitates malignancy in non-small-cell lung cancer cells via regulating LASP1. Respir Res, 2020, 21(1): 235. doi: 10.1186/s12931-020-01464-3 PMC7493870 32938459

[26] Zhang ML, Zhao TT, Du WW, et al. C-MYC-induced upregulation of LINC 01503 promotes progression of non-small cell lung cancer. Eur Rev Med Pharmacol Sci, 2020, 24(21): 11120-11127. doi: 10.26355/eurrev_202011_23599 33215429

[27] Wang H, Jia X, Zhang M, et al. Isoliquiritigenin inhibits virus replication and virus-mediated inflammation via NRF2 signaling. Phytomedicine, 2023, 114: 154786. doi: 10.1016/j.phymed.2023.154786 37002973

[28] Zhang Z, Yung KK, Ko JK. Therapeutic intervention in cancer by isoliquiritigenin from licorice: a natural antioxidant and redox regulator. Antioxidants (Basel), 2022, 11(7): 1349. doi: 10.3390/antiox11071349 PMC9311861 35883840

[29] Kim DH, Park JE, Chae IG, et al. Isoliquiritigenin inhibits the proliferation of human renal carcinoma Caki cells through the ROS-mediated regulation of the Jak2/STAT3 pathway. Oncol Rep, 2017, 38(1): 575-583. doi: 10.3892/or.2017.5677 28560439

[30] Zhang Z, Chen WQ, Zhang SQ, et al. Isoliquiritigenin inhibits pancreatic cancer progression through blockade of p 38 MAPK-regulated autophagy. Phytomedicine, 2022, 106: 154406. doi: 10.1016/j.phymed.2022 36029643

[31] He J, Deng Y, Ren L, et al. Isoliquiritigenin from licorice flavonoids attenuates NLRP3-mediated pyroptosis by SIRT6 in vascular endothelial cells. J Ethnopharmacol, 2023, 303: 115952. doi: 10.1016/j.jep.2022.115952 36442759

[32] Wang JR, Luo YH, Piao XJ, et al. Mechanisms underlying isoliquiritigenin-induced apoptosis and cell cycle arrest via ROS-mediated MAPK/STAT3/NF-kappaB pathways in human hepatocellular carcinoma cells. Drug Dev Res, 2019, 80(4): 461-470. doi: 10.1002/ddr.21518 30698296

[33] Chen XY, Ren HH, Wang D, et al. Isoliquiritigenin induces mitochondrial dysfunction and apoptosis by inhibiting mitoNEET in a reactive oxygen species-dependent manner in A 375 human melanoma cells. Oxid Med Cell Longev, 2019, 2019: 9817576. doi: 10.1155/2019/9817576 30805086 PMC6360568

[34] Huang Y, Liu C, Zeng WC, et al. Isoliquiritigenin inhibits the proliferation, migration and metastasis of Hep3B cells via suppressing cyclin D1 and PI3K/AKT pathway. Biosci Rep, 2020, 40(1): BSR20192727. doi: 10.1042/BSR20192727 PMC6944659 31840737

[35] Wang C, Chen Y, Wang Y, et al. Inhibition of COX-2, mPGES-1 and CYP4A by isoliquiritigenin blocks the angiogenic Akt signaling in glioma through ceRNA effect of miR-194-5p and lncRNA NEAT1. J Exp Clin Cancer Res, 2019, 38(1): 371. doi: 10.1186/s13046-019-1361-2 PMC6704644 31438982

